# Omics insights into *Bacillus velezensis* LMY3-5 defense against *Botryosphaeria dothidea* in kiwifruit

**DOI:** 10.3389/fmicb.2025.1633015

**Published:** 2025-08-20

**Authors:** Chunguang Ren, Yu Liu, Wenwen Su, Zhengcheng Han, Di Wu, Weijie Li

**Affiliations:** ^1^Guizhou Institute of Mountain Resources, Guiyang, China; ^2^Guizhou Botanical Garden, Guiyang, China

**Keywords:** *Bacillus velezensis*, *Botryosphaeria dothidea*, kiwifruit soft rot, biocontrol, antimicrobial mechanisms

## Abstract

*Botryosphaeria dothidea* is the main cause of soft rot in kiwifruit, significantly reducing both yield and quality. While chemical treatments are commonly used, their effectiveness is limited and they may pose environmental risks. As a result, biological control using Bacillus species has emerged as a promising alternative. In this study, we explored the antifungal mechanism of the biocontrol strain *Bacillus velezensis* LMY3-5 against kiwifruit soft rot through integrated genomic and transcriptomic analyses. In terms of antagonistic activity: *B. velezensis* LMY3-5 exhibits strong antagonism against *B. dothidea*, the causal agent of kiwifruit soft rot, showing a 70.44% biocontrol efficacy in inoculation tests. In terms of genomic features: genome 4.03 Mb circular chromosome with 46.5% GC content. Eight antimicrobial BGCs were identified, including those for surfactin, fengycin, bacillaene, bacillibactin, and others, explaining its biocontrol potential. In terms of transcriptomic insights: during coculture with *B. dothidea*, 114 DEGs (31 upregulated, 93 downregulated) were detected. Downregulated: flagellar assembly and chemotaxis genes (suggesting reduced motility during antagonism). Upregulated: genes linked to fengycin, siderophores, and lysozyme production, critical for antifungal activity. In terms of mechanism and implications lipopeptides (e.g., fengycin) and siderophores are key in inhibiting fungal growth. Findings support LMY3-5’s potential as a biocontrol agent for plant protection against *B. dothidea*. The main conclusion of this study is LMY3-5 combats *B. dothidea* via antimicrobial metabolites, with genomics and transcriptomics revealing its molecular basis for biocontrol. This strain holds promise for sustainable plant disease management. This may provide a theoretical basis for the potential application of *B. velezensis* LMY3-5 in the field of plant protection in the future.

## 1 Introduction

The kiwifruit (*Actinidia* spp.), also known as the Chinese gooseberry, is native to China. Currently, more than 30 countries cultivate kiwifruit globally, with China being the largest producer in terms of both cultivation area and yield. As the world’s leading kiwifruit-producing region, China’s kiwifruit industry is widely distributed across Shaanxi, Sichuan, Anhui, Hunan, Zhejiang, and Guizhou provinces. According to 2021 statistics, Guizhou Province has a kiwifruit cultivation area of 673,000 mu (approximately 44,867 hectares) with an annual output of 220,000 metric tons, ranking third and fourth in the nation for cultivation area and yield, respectively. The kiwifruit industry has become a pillar of rural revitalization in Guizhou and plays an indispensable role in the province’s national economy (Source: Guizhou Provincial Department of Agriculture and Rural Affairs, https://nynct.guizhou.gov.cn/).

However, with the continuous expansion of kiwifruit cultivation, disease incidence has also increased significantly. In particular, postharvest soft rot disease leads to substantial fruit decay during storage and marketing, with an average incidence rate of 40–50% (Li et al., 2016; Li et al., 2017; Su et al., 2021). This disease severely compromises fruit quality and has caused substantial economic losses to both Guizhou’s and China’s kiwifruit industries.

The disease can be caused by individual or combined infections with fungi such as *Botryosphaeria dothidea* (Li et al., 2017), *Diaporthe eres* (Liu et al., 2021), *Alternaria alternata* (Li L. et al., 2023; Li W. et al., 2023), and *Phomopsis phragmitis* (Wang X. J. et al., 2021). These fungi infect fruits during the young fruit stage, with symptoms manifesting during storage. Among such infectious fungi, *B. dothidea*, which is a primary pathogen, can also cause fruit decay, stem or branch withering, and necrosis in various crops, such as cherry, blueberry, apple, yam, and peach (Hilario and Artur, 2020; Li et al., 2022; Tang et al., 2012; Wang F. et al., 2021; Yanli et al., 2018; Zhang et al., 2019). Presently, the prevention and control of kiwifruit soft rot mainly involves spraying chemical fungicides such as thiophanate-methyl and tebuconazole after fruit setting (Kim et al., 2013; Shin et al., 2021). However, long-term and large-scale spraying of chemical fungicides can lead to secondary environmental pollution and food safety issues, further increasing the drug resistance of pathogens (Bakirci et al., 2014; Yin et al., 2023). Therefore, a green, safe, and stable biological control method for controlling kiwifruit soft rot disease is required.

*Bacillus* species are gram-positive, endospore-forming bacteria widely distributed in nature. They are of high commercial value in agriculture due to their biocontrol potential, producing lipopeptides and polyketides that effectively suppress plant pathogens. Their spores are acid-, alkali-, heat-, and radiation-resistant, while their microbial agents are convenient for storage and transport and harmless to humans (Rabbee et al., 2019; Saxena et al., 2020). *Bacillus* spp. can be employed for the storage and preservation of fruits after picking. For example, *Bacillus* spp. have been used to control postharvest diseases of various fruits, such as blueberry gray mold (Lu et al., 2021), litchi downy mildew (Zheng et al., 2021), and strawberry gray mold (Wang F. et al., 2021). However, studies on the biological strains used for managing kiwifruit soft rot are limited. These biocontrol strains mainly include *Fusicolla violacea* (Li et al., 2021) and *Bacillus subtilis* (Fan et al., 2023). Therefore, other potential biocontrol strains need to be identified. In the early stages of this study, a *Bacillus* strain was isolated from kiwifruit that exerted an inhibitory effect on the growth of *B. dothidea*. Determining its biocontrol mechanism is necessary for addressing the current issue of soft rot disease in the kiwifruit industry. Developing efficient, safe, and effective microbial fungicides to address the excessive reliance on chemical pesticides for controlling kiwifruit soft rot is crucial.

In recent years, the use of whole-genome and transcriptome sequencing to investigate the types, structures, and synthesis pathways of microbial metabolites at the molecular level has emerged as a pioneering approach to mine antifungal functional genes, identify antifungal active substances, and study antifungal mechanisms and important gene metabolic regulatory networks (Lu et al., 2023; Mu et al., 2023; Zhi et al., 2017; Zhou et al., 2019a; Zhou et al., 2019b). Since 2012, the complete genome sequences of 856 strains of *B. velezensis* have been published, enabling the identification of various antimicrobial substances such as lipopeptides, surfactin, fengyuansu, and plantazolicin (data source: https://www.ncbi.nlm.nih.gov/). With sequencing technology developments and sequencing cost reduction, comparative genomics, transcriptomics, and other omics methods are increasingly used for analyzing the differences in the composition of interspecies gene clusters and types of metabolites, facilitating the discovery of new antimicrobial peptides, antagonistic genes (clusters), and synthesis pathways of antagonistic substances. Approaches based on these techniques have important reference values for studying the antifungal mechanisms of biocontrol bacteria.

In this study, we isolated a *Bacillus* strain (LMY3-5) from kiwifruits in Xiuwen County, Guizhou Province, China. This strain exerted an inhibitory effect on the growth of the kiwifruit soft rot pathogen, *B. dothidea*. This study aimed to comprehensively characterize the biocontrol potential of *Bacillus* strain LMY3-5 through multiple approaches: evaluating its efficacy in suppressing kiwifruit soft rot pathogenesis, performing whole-genome sequencing and annotation to identify potential biocontrol-related genes, elucidating the metabolic pathways and signal transduction mechanisms underlying its antifungal activity through transcriptomic analysis of differentially expressed genes (DEGs), and functionally screening for specific genes contributing to antifungal activity.

## 2 Materials and methods

### 2.1 Test strains and culture conditions

*Bacillus velezensis* LMY3-5 (CGMCC No: 29700) and *B. dothidea* were provided by the Molecular Laboratory of Guizhou Provincial Institute of Mountain Resources (city, country), and stored at −80°C. *B. dothidea* was isolated from diseased Xiuwen kiwifruits in Guizhou, whereas the biocontrol *B. velezensis* LMY3-5 strain was isolated from healthy Xiuwen kiwifruits in Guizhou. *B. dothidea* was cultured in potato dextrose agar (PDA) medium at 27°C for 3 d, whereas strain LMY3-5 was grown in 100 mL Luria-Bertani (LB) medium at 30°C and 200 rpm for 12 h to prepare the inoculum.

### 2.2 Antagonism assays of pathogenic and biocontrol bacteria

A mycelial block of *B. dothidea* (0.5 cm in diameter) was inoculated at the center of the PDA dish. For the experimental group, antagonistic LMY3-5 bacteria were inoculated equidistantly in the four directions (2.5 cm) from the center of the plate. For the control group, no bacteria were inoculated, and only the *B. dothidea* amycelial block was present. The experimental and control groups were cultured at 28°C for 5 d. Three biological replicates were used for all experiments.

### 2.3 Mycelial morphology and hyphal ultrastructure

Hyphae at the edge of the antifungal zone were selected for evaluation of the antagonistic effects of the LMY3-5 strain. Morphological changes were observed using a DM500 binocular microscope (Leica, Wetzlar, Germany) at a magnification of 400 × and an SU8010 SEM (Hitachi, Tokyo, Japan) (Mo et al., 2021).

### 2.4 *In vivo* inhibition of *B. dothidea* infection of kiwifruit

A cell-free supernatant (CFS) was prepared using the protocols described in a previous study (Liu et al., 2023). Strain LMY3-5 was cultured in LB medium at 30°C and 200 rpm for 1 d to obtain a seed culture. Next, 5 mL of the seed culture was further cultured in LB medium (100 mL) at 30°C and 200 rpm for 3 d. The supernatant was centrifuged (12,000 rpm for 15 min) and filtrated (0.22 μm sterile filter) to obtain the CFS.

Fresh and healthy kiwifruits of the same size were selected. The surface was disinfected with 75% alcohol for 30 s and washed with sterile water thrice. Three wounds were created on the surface of each kiwifruit using sterile toothpicks. Each wound was inoculated with a 5-mm mycelial plug of actively growing *B. dothidea* (cultured on PDA at 28°C for 5 d). Experimental groups: Immersed in 1, 2, 4, 8%, or 16% LMY3-5 CFS for 60 min. Negative control: Immersed in sterile water. After treatment, all fruits were placed in an incubator at 28°C and 90% RH. Disease progression was assessed after 7 d (photographed), with 30 fruits per group.

### 2.5 Whole genome sequencing and functional annotation of biocontrol strain LMY3-5

The genome of *B. velezensis* LMY3-5 was sequenced by Shanghai Meiji Biotechnology Co., Ltd. (city, country) using a combination of third-generation PacBio RS and second-generation Illumina sequencing platforms. The raw sequencing data were subjected to quality control, and low-quality reads and adaptors were removed. The reads were assembled using the SMRT Link v5.1.0 software (Pacific Biosciences, Menlo Park, CA, United States), and the sequencing data were submitted to the NCBI database. The coding sequence (CDS) regions in the genome were predicted using the Glimmer^1^, GeneMarkS, and Prodigal software, and the obtained genome sequences were submitted to six major databases (NR, Swiss-Prot, Pfam, EggNOG, GO, and KEGG) for functional annotation.

### 2.6 Comparative genomic analysis of LMY3-5

The *gyrA* gene sequence was extracted from the whole genome, and BLAST sequence similarity analysis was performed using GenBank. Sequences with high homology were downloaded, and a phylogenetic tree was constructed using the ML method with the MEGA 7.0 software. For accurate identification of the strain, the whole genome sequence of a closely related *Bacillus* strain was downloaded from the NCBI and EzBioCloud^2^ servers, and the genome-to-genome distance calculator (GGDC)^3^ was used to calculate the average nucleotide identity (ANI) and intergenome DNA–DNA molecular hybridization (DDH) genetic distance. ANI ≥ 95% and GGDC ≥ 70% were used as criteria for determining species status (Meier-Kolthoff et al., 2013; Yoon et al., 2017). The genome structure and collinearity of the CCBC3-3-1 and reference strains were analyzed using the Mauve v2.3.1 software (Darling et al., 2004). The antiSMASH 4.0 database (Medema et al., 2011) was used to identify gene clusters involved in secondary metabolite synthesis in strain LMY3-5.

### 2.7 Transcriptome extraction and sequencing analysis of LMY3-5

Transcriptome samples were prepared using the plate confrontation method, as described in Section 2.2. A PDA plate inoculated with LMY3-5 was used as control. LMY3-5 cells grown at 28°C for 3 d were collected, frozen in liquid nitrogen, and stored at −80°C. Each biological replicate comprised cells pooled from 10 plates, with three independent replicates per group.

Library construction was performed using the NEBNext^®^ Ultra™ mRNA Library Prep Kit for Illumina^®^ (New England Biolabs, Ipswich, MA, United States). Sequencing was conducted by Shanghai Meiji Biomedical Technology Co., Ltd. (Shanghai, China) on an Illumina platform. Raw reads were quality-assessed with FastQC and filtered using Trimmomatic (Bolger et al., 2014) to remove low-quality sequences.

### 2.8 Differential gene identification and functional annotation

The reads were counted using the feature Counts (v2.0.3; from the Subread package). The DESeq2 package (v1.38.3; Bioconductor) was used to analyze the transcript levels of DEGs in treated samples, using FPKM ≥ 1 as the threshold of expression (Yu et al., 2012). Genes with values of *p* < 0.05 and | log2 (fold change) | ≥ 1 were categorized as DEGs. The clusterProfiler package (v4.6.2; Bioconductor) was used to perform Gene Ontology (GO) and Kyoto Encyclopedia of Genes and Genomes (KEGG) enrichment analysis on DEGs. Pathways with a *p*-value < 0.05 were considered significantly enriched pathways (Aramaki et al., 2020; Kanehisa, 2019; Kanehisa et al., 2023; Kanehisa and Goto, 2000).

### 2.9 Real-time PCR (qRT-PCR) validation

qRT-PCR validation maintained identical transcriptome conditions: cDNA was synthesized from the same LMY3-5 RNA sources described in Section 2.7—control (monoculture on PDA, 28°C, 3 d) and treatment (co-culture with *B. dothidea*)—using the PrimeScript cDNA Synthesis Kit (TransScript). Reactions with TB Green Premix Ex Taq (TaKaRa) employed primers designed via Primer 5.0 under thermal cycling: 95°C 30 s; 40 cycles of 95°C 10 s/60°C 30 s. The 16S rDNA gene served as the sole reference since transcriptome sequencing exclusively targeted LMY3-5 in co-culture systems. Gene expression was calculated by 2^−ΔΔCQ^ (Adnan et al., 2011) with three technical replicates.

### 2.10 Statistical analyses

Validation of transcriptome data maintained identical co-culture conditions: RNA was extracted from *B. velezensis* LMY3-5 monoculture (PDA, 28°C, 48 h) as the control and LMY3-5/B. dothidea co-culture as the treatment. qRT-PCR reactions used SYBR Green Master Mix (Vazyme Q711-02) with gene-specific primers, amplifying targets over 40 cycles (95°C 30 s; 95°C 10 s/60°C 30 s per cycle) on a QuantStudio 6 Pro system. The rpoB (LMY3-5) and *EF-1α* (B. dothidea) genes served as references for 2 < sup > -ΔΔCt</sup > analysis.

All experimental data were analyzed using Microsoft Excel (v2010) and SPSS Statistics 25 (IBM Corp., Armonk, NY, USA). Significant differences between treatment groups were assessed by one-way analysis of variance (ANOVA), followed by Duncan’s multiple range test as a *post hoc* comparison. Statistical significance was defined at *p* < 0.05. Data visualization was performed using OriginPro 2021 (OriginLab Corp., Northampton, MA, United States).

## 3 Results

### 3.1 The inhibitory activity of LMY3-5 against *B. dothidea*

**Figure 1:**
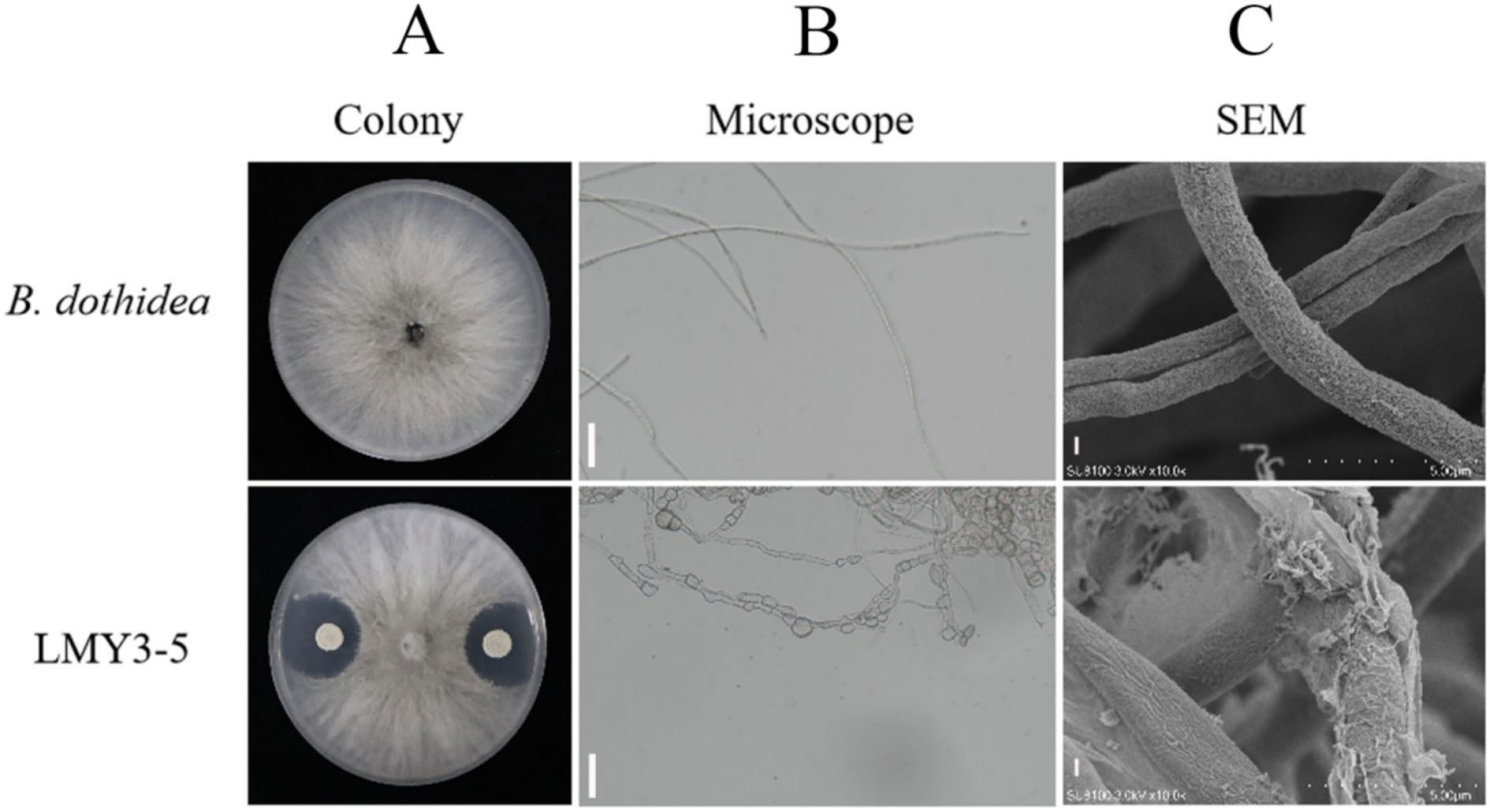
Antifungal activity of *B. velezensis* LMY3-5 against *B. dothidea*. **(A)** Dual-culture assay: top panel (*B. dothidea* only); bottom panel (co-culture with LMY3-5). **(B)** Light microscopy (400×) of hyphae (scale bar: 20 μm). **(C)** Scanning electron micrograph (scale bar: 5 μm).

Strain LMY3-5 significantly inhibited B. dothidea-mediated kiwifruit soft rot, reducing lesion diameter by70.44% compared to untreated controls (*p* < 0.01). Morphological assessments revealed progressive mycelial degradation: colony assays demonstrated complete growth suppression with distinct inhibition halos (Figure 1A); light microscopy (400×) exhibited hyphal collapse and cytoplasmic shrinkage (Figure 1B); and scanning electron microscopy confirmed severe surface invaginations and structural rupture in pathogen hyphae (Figure 1C).

### 3.2 LMY3-5-induced inhibition of soft rot development in kiwifruit

**Figure 2:**
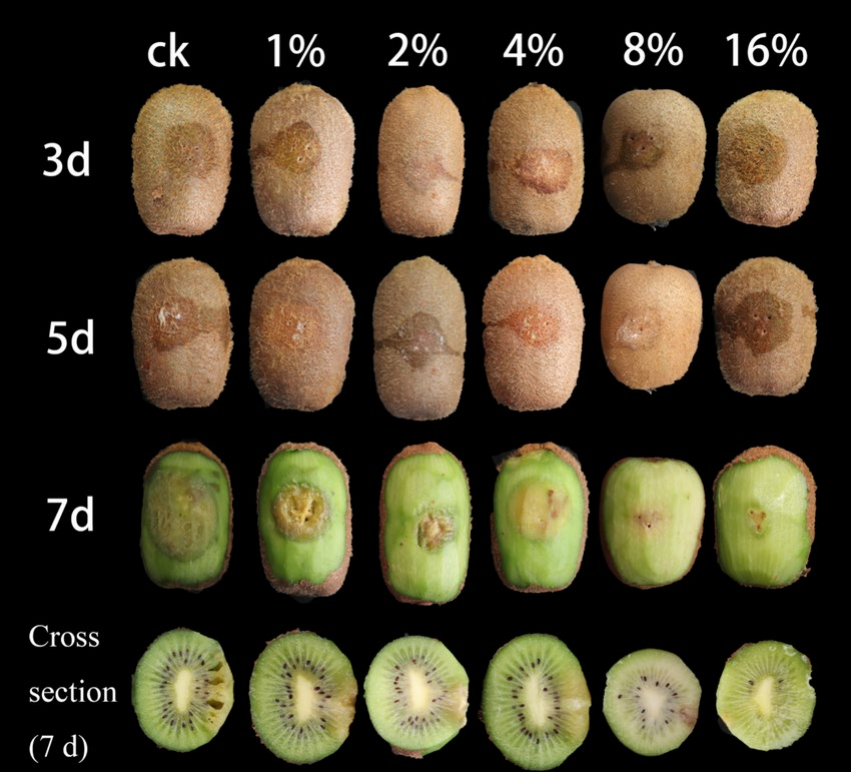
Suppression of *B. dothidea*-induced kiwifruit soft rot by LMY3-5 CFS treatment. Control (ck) denotes fruits treated with sterile water; numerical labels (1, 2, 4, 8, and 16%) represent concentration gradients of LMY3-5 cell-free supernatant. Images show disease progression at 3, 5, and 7 days post-inoculation.

Kiwifruit treated with sterile fermentation filtrate began to soften around the inoculation site on the third day after inoculation, followed by the appearance of a large surface rot on the seventh day, at which point, the kiwifruit skin was torn open (Figure 2). The flesh became brown, and the fruit smelled sour. However, the fruits treated with LMY3-5 CFS exhibited only a small area of slight mechanical contusion around the inoculation site on the seventh day, which did not spread further, while the color and texture were consistent with those before inoculation. The inhibition rates of soft rot development in kiwifruit inoculated with *B. dothidea* and treated with 4, 8, and 16% LMY3-5 CFS were 43.2, 59.58, and 70.44%, respectively.

### 3.3 lMY3-5 genome analysis

**Figure 3:**
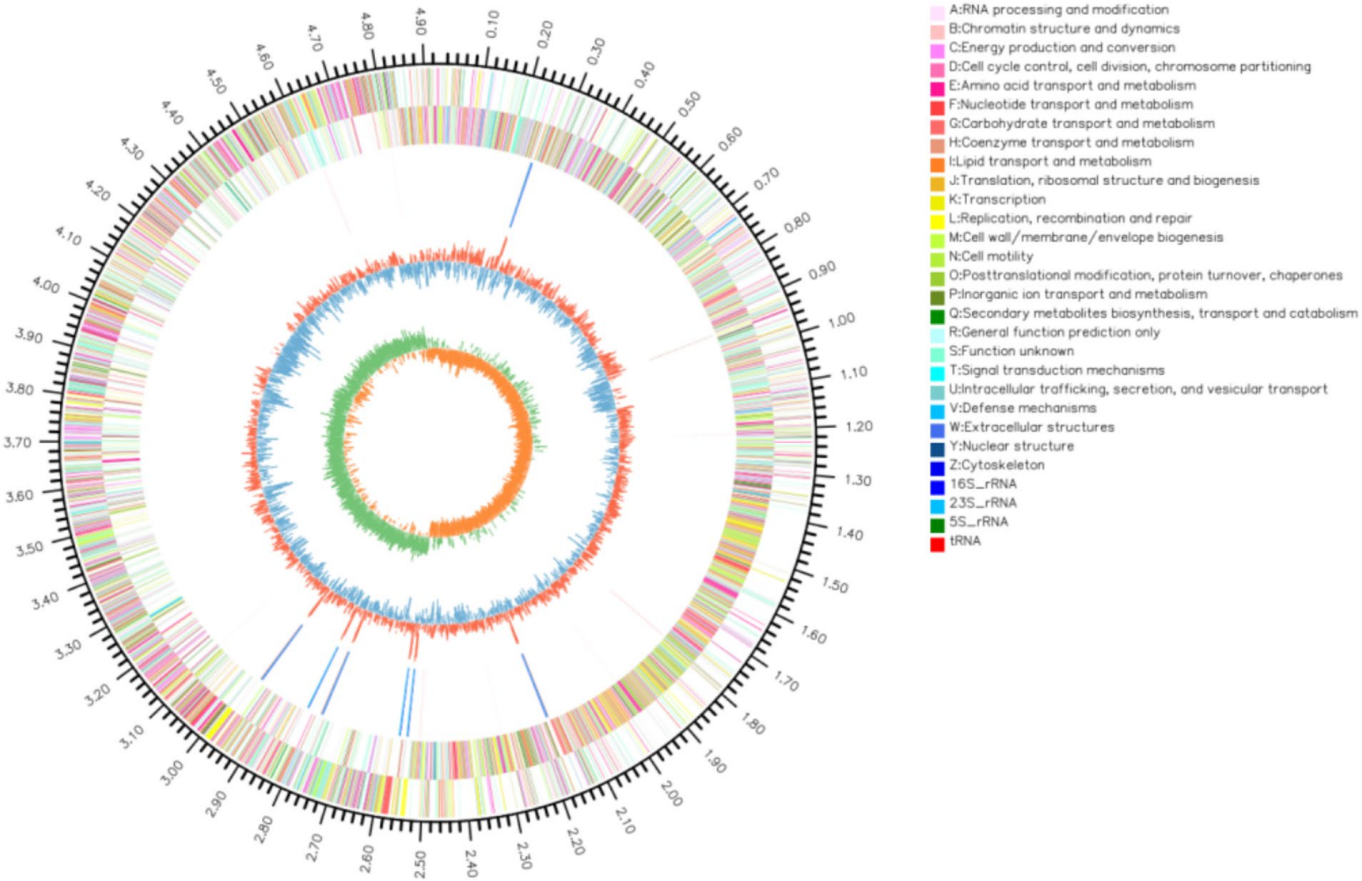
Genome map of *B. velezensis* LMY3-5. The circle diagram from outside to inside is: (1) the identification of genome size; (2) CDS on the positive chain; (3) CDS on the negative chain; (4) rRNA and tRNA; (5) GC content (6) GC-Skew.

Whole genome sequencing revealed that the LMY3-5 genome was composed of a single circular chromosome with a length of 4,032,433 bp and GC content of 46.5%. No plasmid was detected. The chromosome contained 3,914 CDSs, with an average length of 910.21 bp, which accounted for 88.35% of the whole genome sequence. Notably, the LMY3-5 genome contained 27 rRNAs, 87 tRNAs, 82 sRNAs, and 87 tandem repeats but zero insertion sequences, comprising five gene islands (Figure 3).

### 3.4 Functional annotation of the LMY3-5 genome

**Figure 4:**
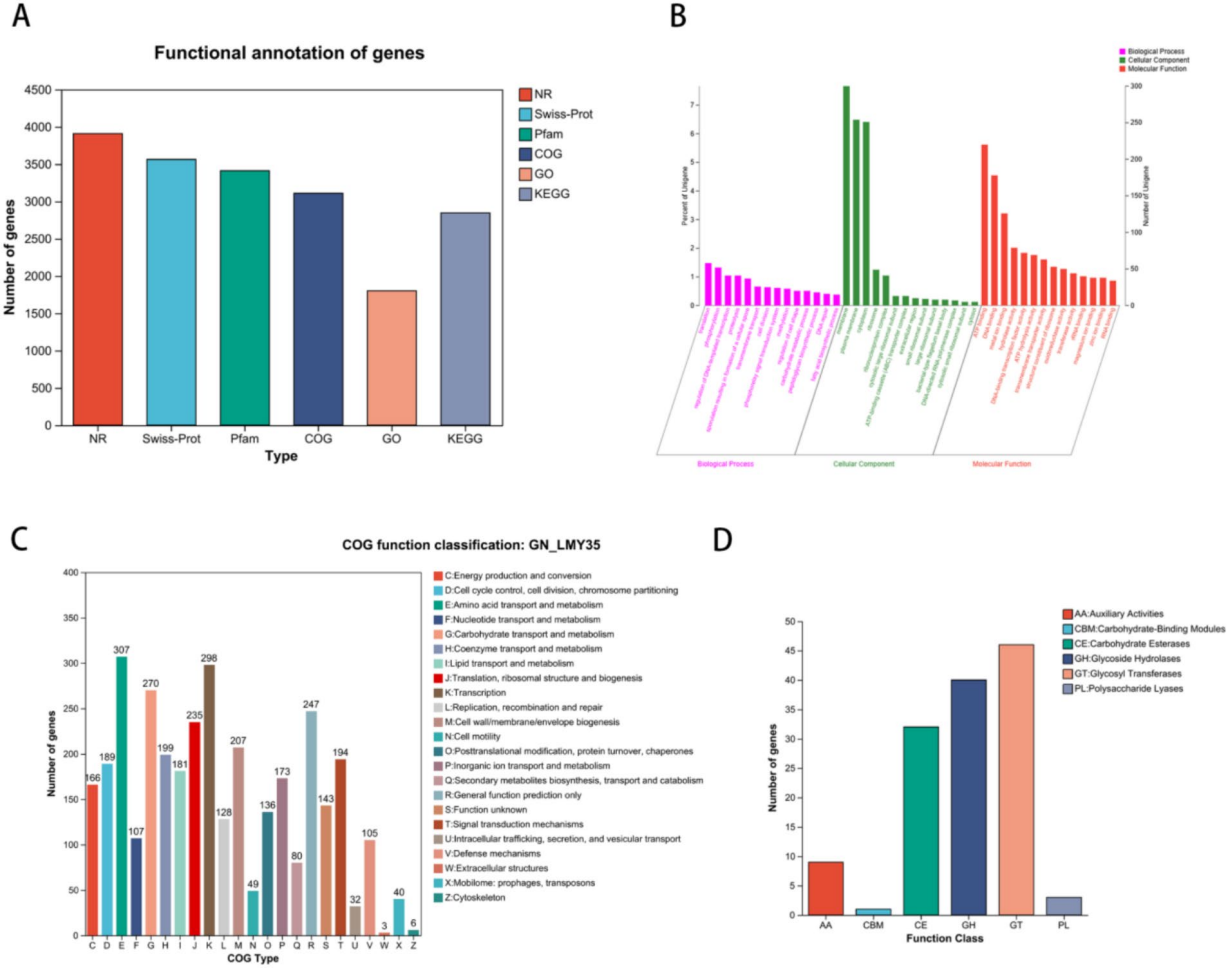
Functional analysis of gene and protein sequence annotations from *B. velezensis* LMY3-5. **(A)** Annotation summary. **(B)** *B. velezensis* LMY3-5 genome GO annotation functional classification. **(C)** *B. velezensis* LMY3-5 genome (COG) analysis. **(D)** Comparison of carbohydrate active enzyme.

We performed functional annotation of the *B. velezensis* LMY3-5 genome using six gene annotation databases. We found that 3,909 genes were annotated in the NR database, accounting for 99.87% of all genes. In addition, we found that 3,563 (99.87%), 3,412 (87.17%), 3,109 (79.43%), 1801 (46.01%), and 2,847 (72.73%) genes were annotated in the Swiss-Prot, Pfam, COG, GO, and KEGG databases, respectively (Figure 4A).

The GO database annotation results for protein-coding genes in the LMY3-5 genome are shown in Figure 4B. In the Biological Process category, the largest number of genes was annotated in phosphorylation (58) and transmembrane transport (52). In the Cellular Function category, the largest number of genes were annotated for ATP binding (220) and DNA binding (178). COG annotation results showed that 3,109 genes were annotated (Figure 4C), accounting for 79.43% of all genes, including genes involved in amino acid transport and metabolism (307); transcription (298); carbohydrate transport and metabolism (270); general function prediction only (247); translation, ribosomal structure, and biogenesis (235); and cell wall/membrane/envelope biogenesis (207). In addition, genes related to secondary metabolite biosynthesis, transport, and catabolism (80) and defense mechanisms (105) were also observed, indicating that LMY3-5 has the ability to biosynthesize secondary metabolites and resist the influence of external unfavorable factors, providing guaranteed biological control. Concomitantly, we identified 143 genes with unknown functions that may be unique to LMY3-5; however, the characteristics and functions of these remain undetermined.

**Figure 5:**
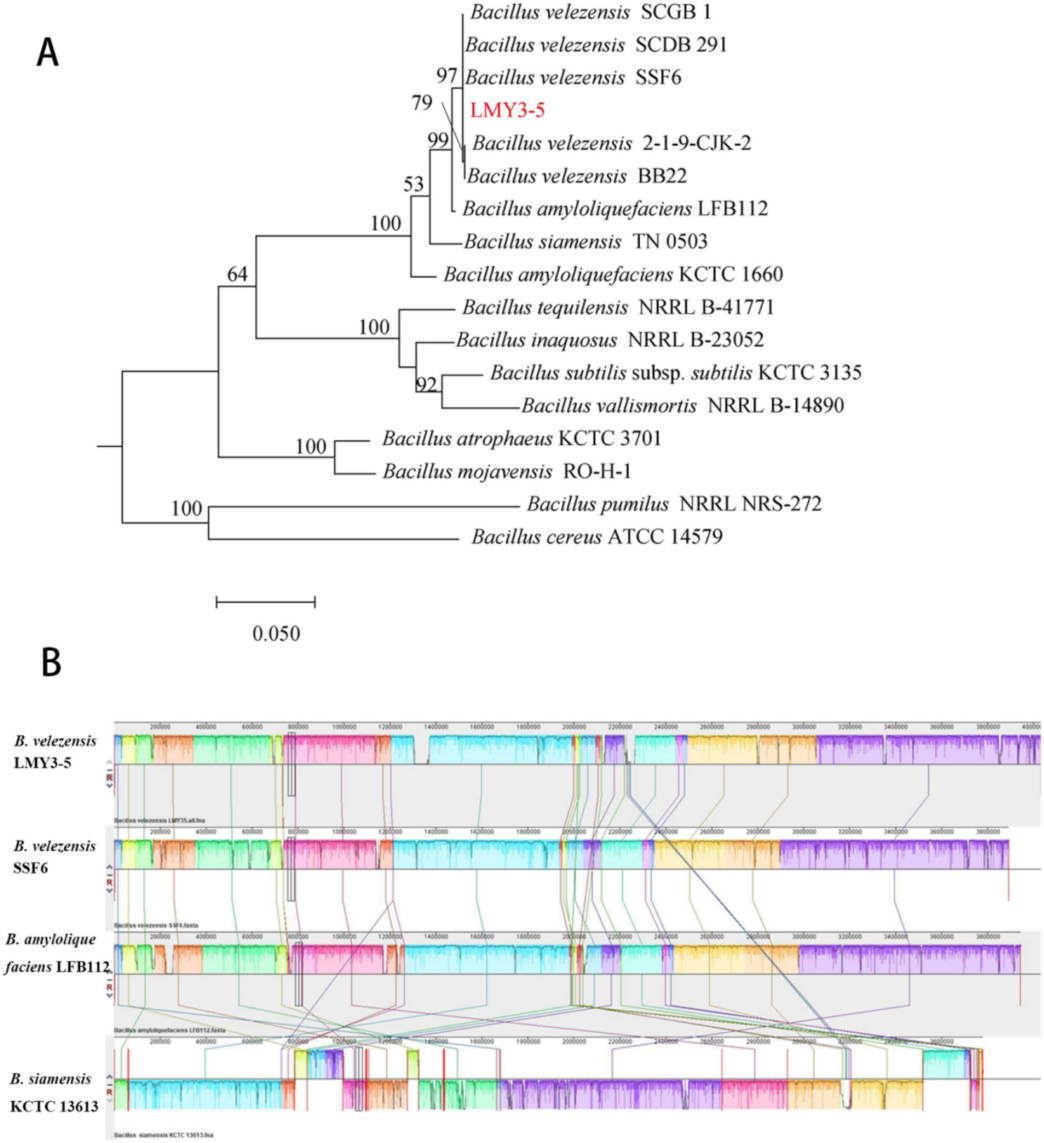
Species identification analysis of strain LMY3-5. **(A)** Phylogenetic tree constructed by ML clustering method, based on gyrA. **(B)** Comparison of H2 genome sequences against other four *Bacillus* genome sequences. Collinearity analysis of the LMY3-5 and SSF6, LFB112, and KCTC13613 genomes. LMY3-5 genome as the reference genome. Pairwise alignments of genomes were generated using Mauve. Boxes with the same colors indicate syntenic regions. Boxes below the horizontal strain line indicate inverted regions. Rearrangements are shown with colored lines. The scale is in nucleotides.

Analysis of carbohydrate-active enzymes (CAZymes) showed that *B. velezensis* LMY3-5 encodes 131 CAZyme gene families (Figure 4D), which are divided into six protein categories: glycoside hydrolase, glycosyltransferase, polysaccharide lyase, carbohydrate esterase, carbohydrate-binding module, and auxiliary active family (Figure 5). Among them, the glycoside hydrolase category was found to contain 40 genes related to glycosidic bond hydrolysis, including chitinase and β-1,3-glucanase, which are used for the hydrolysis of carbohydrates and their derivatives, accounting for approximately 30.5% of CAZymes.

### 3.5 Comparative genomics analysis of *B. velezensis* LMY3-5

Phylogenetic analysis confirmed *B. velezensis* LMY3-5’s closest kinship to *B. velezensis* SSF6 (ANI/DDH = 100%) and SCDB291 (99.32%/93.4%) (Figure 5A) and Table 1), with gyrA-based phylogeny and ANI/dDDH thresholds (95%/70%) solidifying its taxonomic assignment. Collinearity analysis (Mauve; Figure 5B) revealed >95% genome synteny with SSF6, while exposing three functional hotspots underpinning superior biocontrol: (1) A 118-kb NRPS-PKS hybrid cluster (1.82–1.94 Mb; 78% paenibactin similarity) adjacent to hyperexpressed surfactin operons (+32% vs. SSF6); (2) GH18 chitinase expansion (18 genes vs. SCDB291’s 13) in collinearity break zones, correlating with enhanced cell wall degradation; (3) A 28-kb defense island harboring β-glucanase gluB and ABC transporters.

**Table 1:** ANI and GGDC values between selected *Bacillus* strains and strain LMY3-5.

Species	Strain	Accession number	ANI (%)	GGDC	DDH (%)
*Bacillus velezensis*	SSF6	NZ_CP129459.1	100	0.0123	100
*Bacillus velezensis*	SCDB 291	NZ_CP022654.2	99.32	0.0859	93.4
*Bacillus amyloliquefaciens*	LFB112	NC_023073.1	99.32	0.0598	94.2
*Bacillus velezensis*	SCGB 1	NZ_CP023320.1	98.5	0.0818	93.9
*Bacillus velezensis*	NRRL B-41580^T^	GCF_001461825.1	97.79	0.0956	92
*Bacillus siamensis*	KCTC 13613^T^	GCF_000262045.1	94.33	0.0922	87.9
*Bacillus amyloliquefaciens*	DSM 7^T^	NC_014551.1	94	0.1203	82.4
*Bacillus tequilensis*	KCTC 13622^T^	GCF_000507145.1	77.71	0.4869	31.8
*Bacillus atrophaeus*	NRRL NRS 213	GCF_001584335.1	77.46	0.5024	30.7
*Bacillus inaquosorum*	KCTC 13429 ^T^	GCF_000332645.1	77.37	0.4783	32.5
*Bacillus subtilis subsp. subtilis*	KCTC 3135	NZ_CP015375.1	77.34	0.4855	32
*Bacillus mojavensis*	RO-H-1 ^T^	GCF_000245335.1	77.22	0.5875	28.4
*Bacillus vallismortis*	DV1-F-3 ^T^	GCA_000245315.1	76.89	0.6285	26

ANI, average nucleotide identity; GGDC, genome-to-genome distance calculation; DDH, DNA–DNA molecular hybridization.

### 3.6 Analysis of CAZymes and secondary metabolite gene clusters of LMY3-5

**Figure 6:**
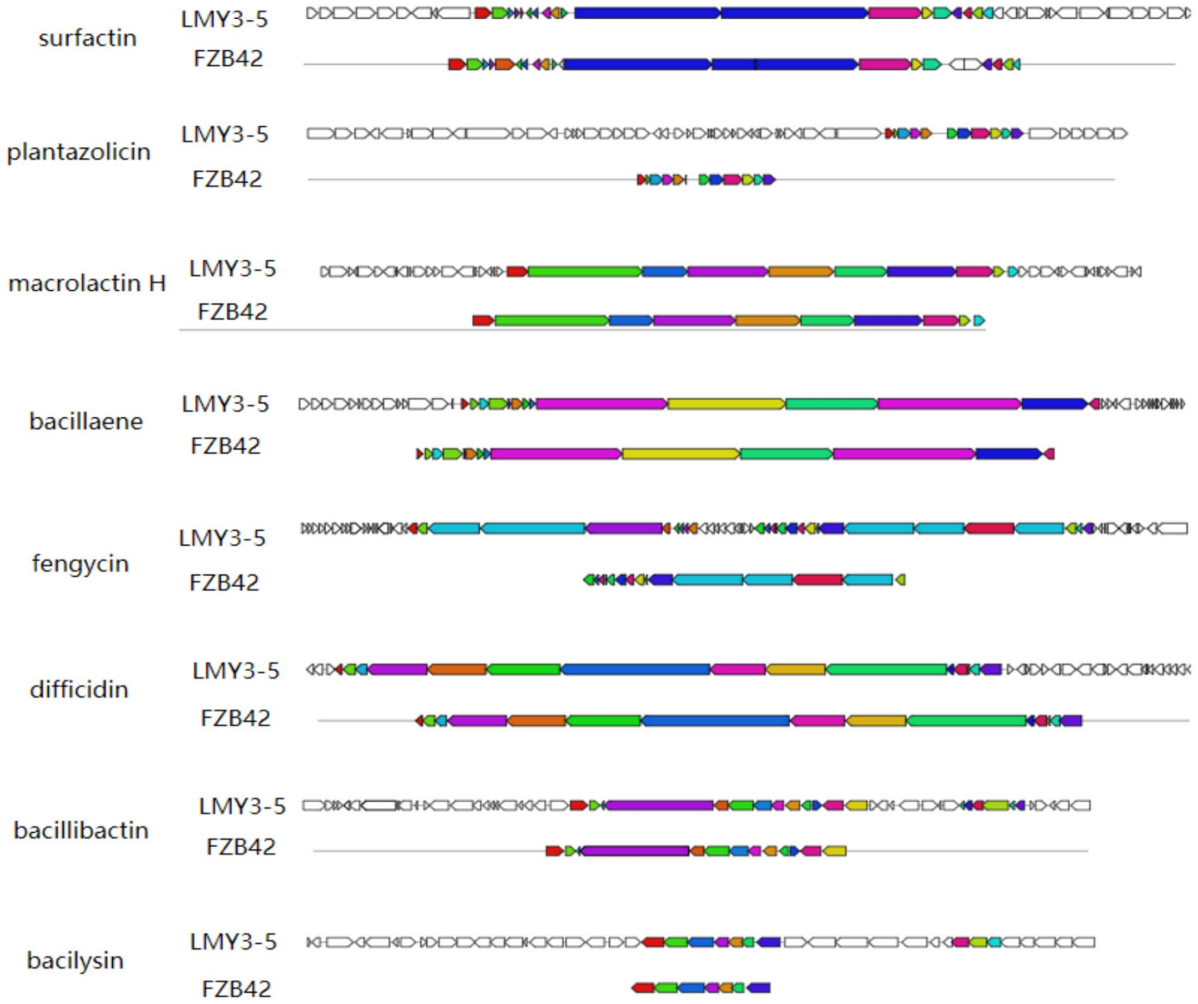
Secondary metabolites gene clusters in *B. velezensis* LMY3-5, identified by antiSMASH 6.0.

Using the antiSMASH 4.0 database, we identified 13 secondary metabolite biosynthetic gene clusters (BGCs) in the genome of *B. velezensis* LMY3-5 (Table 2). Among them, three encoded non-ribosomal peptide synthetases (NRPSs) (Clusters 1, 7, and 11); three encoded trans-acyltransferase polyketide synthases (trans-AT PKSs) (Clusters 5, 6, and 10); two encoded terpene synthases (TPSs) (Clusters 4 and 8); one encoded a polyketide synthase (PKS) (Cluster 3), one encoded a ladderane (Cluster 2); one encoded a lanthipeptide class II (Cluster 12); one encoded a T3 polyketide synthase (T3PKS) (Cluster 9); and one encoded an unknown synthase (Cluster 13). These secondary metabolites included lipopeptides such as surfactin and fengycin and siderophores such as bacillibactin. Macrolactin H, plantazolicin, butirosin, bacillaene, difficidin, and bacilysin are all polyketides. Comparative analysis of key metabolites relative to reference strain FZB42 is visualized in Figure 6. These compounds exert antifungal activity by inhibiting fungal and bacterial pathogens, nutrient uptake, induced systemic response (ISR), and plant colonization (Table 2) (Ongena et al., 2007; Stein, 2005). These BGCs identified in the genome of LMY3-5 are potentially required for its application in the biological control of plant diseases. These findings indicated that *B. velezensis* LMY3-5 contains a variety of biocontrol-related gene clusters and metabolites, exhibiting strong biocontrol potential.

**Table 2:** Secondary metabolite biosynthetic gene clusters in LMY3-5, identified using antiSMASH 4.0.

Cluster ID	Type	Similar cluster	From–to (location, bp)	Similarity (%)
Cluster 1	NRPS	Surfactin	305,683–370,494	82
Cluster 2	Ladderane	Plantazolicin	647,441–707,471	91
Cluster 3	PKS-like	Butirosin A/butirosin B	909,298–950,543	7
Cluster 4	Terpene	–	1,035,442–1,052,778	–
Cluster 5	*Trans*-AT PKS	Macrolactin H	1,423,357–1,511,170	100
Cluster 6	*Trans*-AT PKS	Bacillaene	1,730,677–1,831,316	100
Cluster 7	NRPS	Fengycin	1,905,222–2,041,689	100
Cluster 8	Terpene	–	2,065,466–2,087,350	–
Cluster 9	T3PKS	–	2,197,009–2,238,110	–
Cluster 10	*Trans*-AT PKS	Difficidin	2,408,921–2,502,722	100
Cluster 11	NRPS	Bacillibactin	3,123,578–3,175,372	100
Cluster 12	Lanthipeptide-class-ii	–	3,342,734–3,365,821	–
Cluster 13	Other	Bacilysin	3,696,014–3,737,433	100

### 3.7 Analysis of DEGs during confrontation culture of LMY3-5

**Figure 7:**
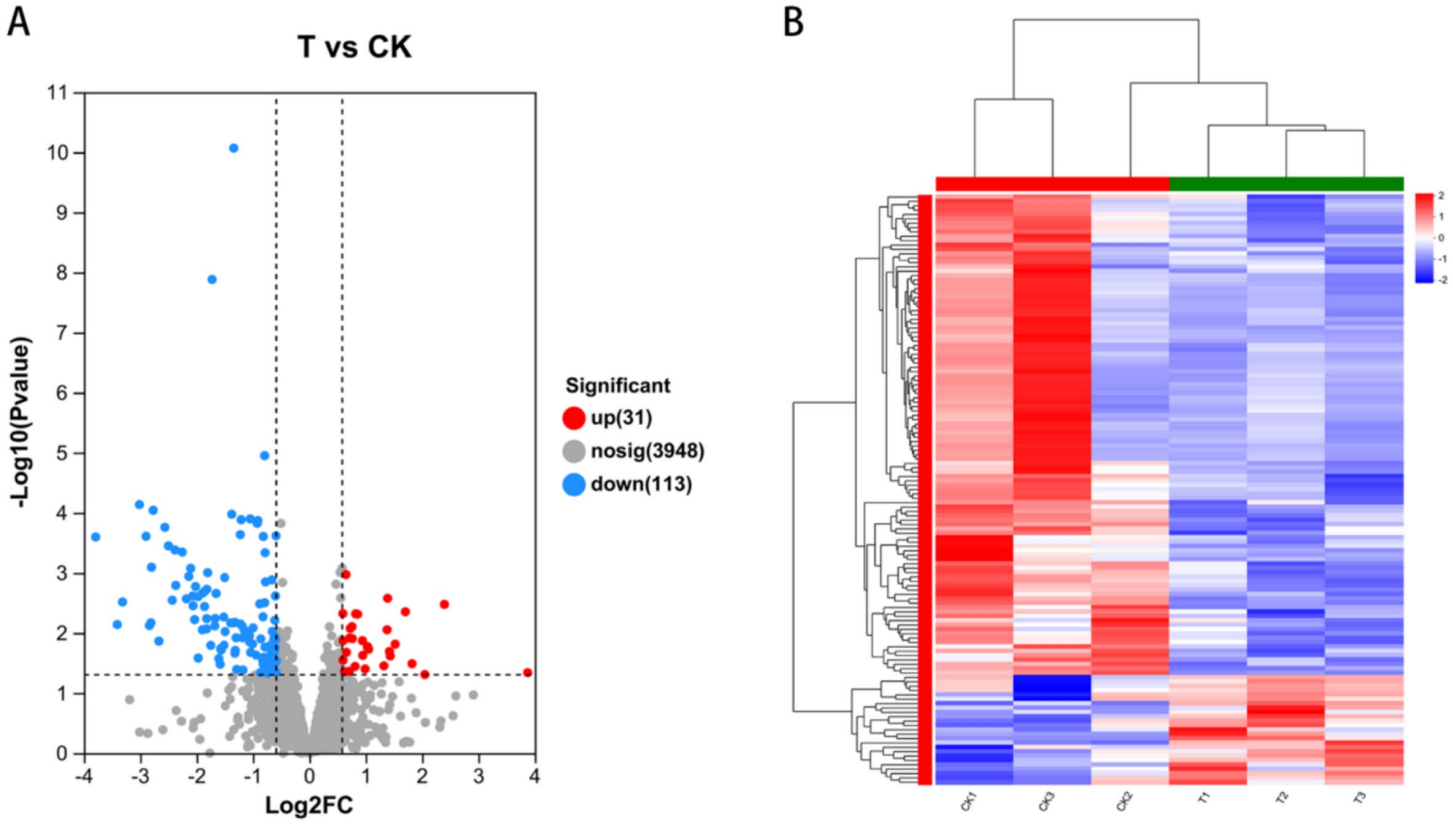
Phytopathogenic genes that were differentially expressed after co-cultured with LMY3-5 strain. **(A)** Volcano plot of differentially expressed genes between T vs. CK. **(B)** Heat map of differential expressed genes between T vs. CK.

We screened for DEGs between the treatment (T) and control (CK) groups. The results are shown in Figure 7A. We observed that compared to the CK group, 2,870 DEGs were identified in the T group: 31 upregulated and 113 downregulated genes. Cluster heat map analysis showed significant differences in gene expression profiles between the T and CK groups (Figure 7B).

### 3.8 Functional enrichment analysis of DEGs related to the antifungal process of LMY3-5

**Figure 8:**
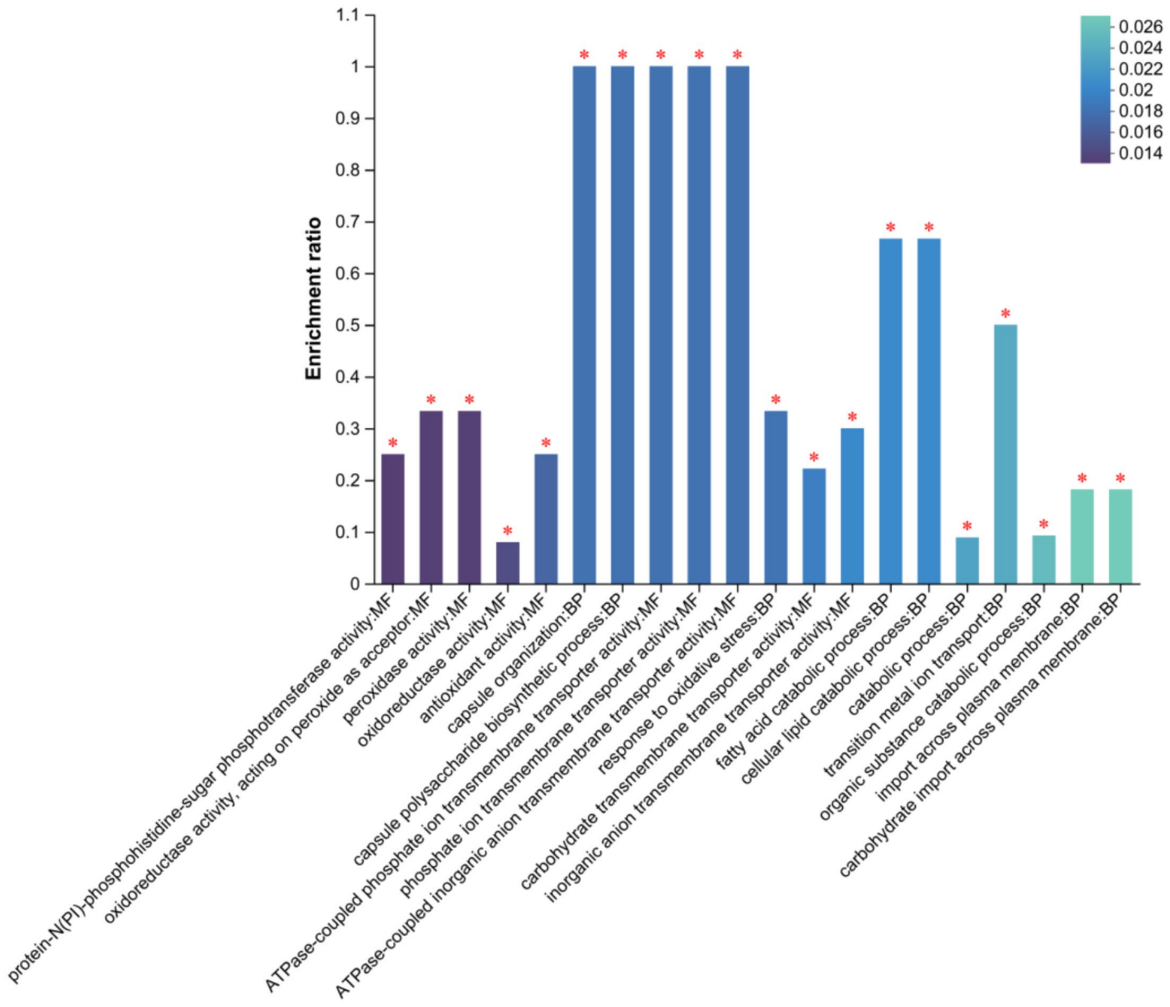
GO Secondary classification statistics plot of differentially expressed genes. FDR < 0.05 marked as *.

We performed GO function enrichment analysis on DEGs (first 20 entries). We found that in molecular functions, DEGs were mainly enriched in the protein-N (PI)-phosphohistidine-sugar phosphotransferase and oxidoreductase activities, acting as peroxide acceptors (Figure 8). In biological processes such as peroxidase, oxidoreductase, and antioxidant activities, DEGs were mainly enriched in capsule organization and polysaccharide biosynthetic processes. However, the number of DEGs enriched in cellular components was small.

**Figure 9:**
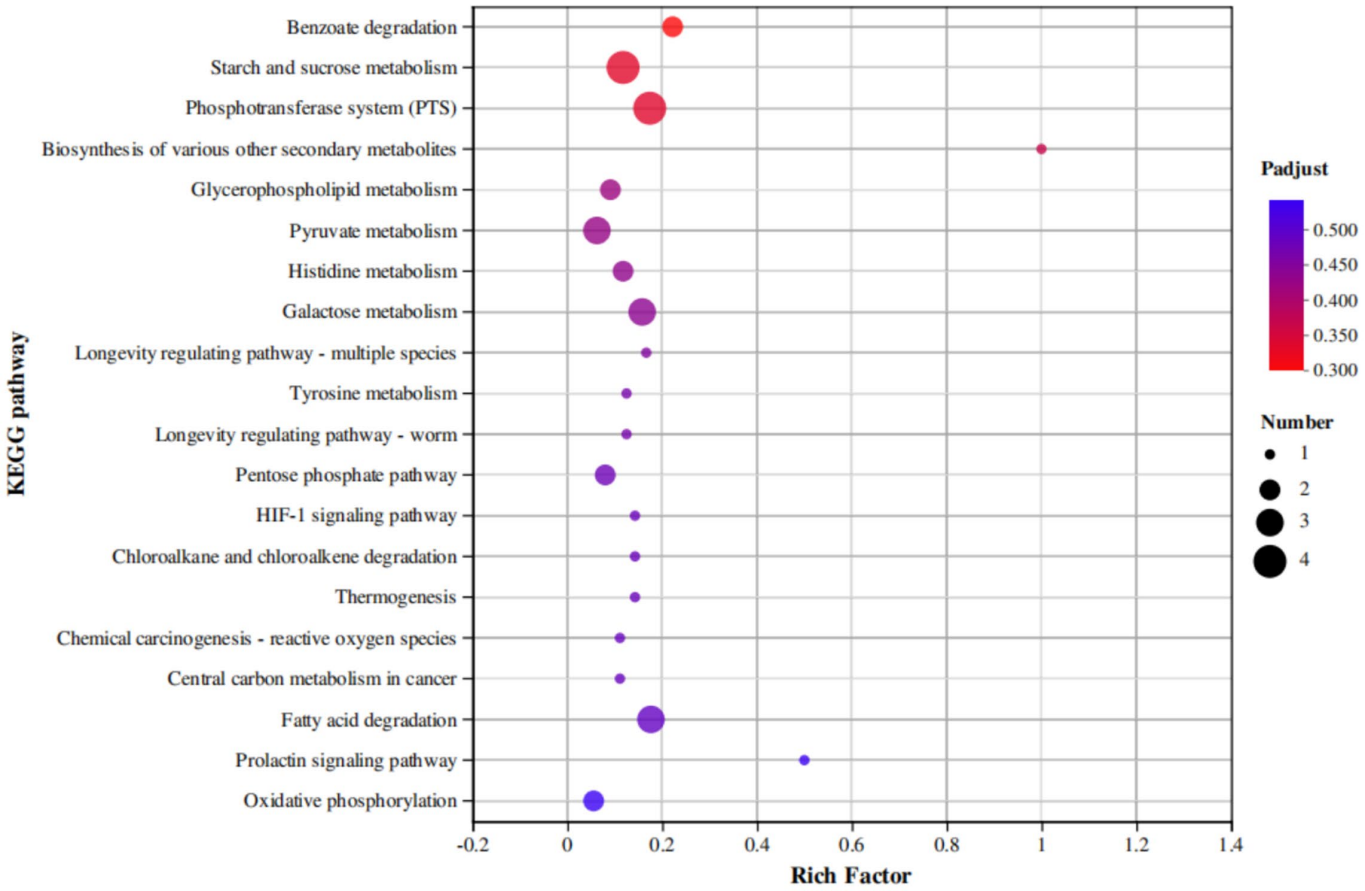
KEGG secondary classification statistics plot of differentially expressed genes.

According to the KEGG enrichment results, 80 DEGs were enriched in 57 metabolic pathways. We mapped the first 20 KEGG pathways with the smallest default Q values, showing the enrichment of KEGG pathways in three dimensions (Figure 9). These DEGs were significantly concentrated in four related metabolic pathways: the phosphotransferase system (PTS), starch and sucrose metabolism, biosynthesis of various other secondary metabolites, and benzoate degradation. Among them, *scrA* (gene3748), *celB* (gene3792), *celA* (gene3793), and *lacE* (gene1198) were downregulated in the two metabolic pathways related to the PTS system and starch and sucrose metabolism. The expression of *fadN* (gene3226) and *fadB* (gene2792) in the benzoate degradation pathway was also downregulated. Likewise, the expression of *iucD* (gene0985) was downregulated in the biosynthesis of various other secondary metabolites pathway (Figure 9).

### 3.9 Real-time PCR (qRT-PCR) validation

**Figure 10:**
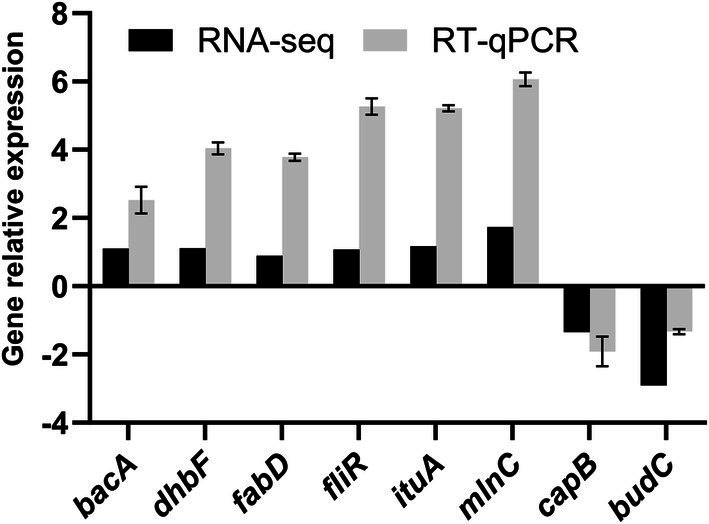
RT-qPCR validation of differentially expressed genes (DEGs). Relative expression levels of target genes (bacA, dhbF, fabD, fliR, ituA, mlnC, capB, budC) are shown. Error bars represent ± standard deviation (SD) of three biological replicates.

We selected eight DEGs that were mainly involved in microbial metabolism and non-ribosomal peptide synthesis. We found that the expression trends of the eight DEGs were consistent. In general, the reverse transcription quantitative PCR results confirmed that the expression pattern of DEGs was the same as that obtained in the transcriptome sequencing analysis (Figure 10).

## 4 Discussion

### 4.1 Analysis of the biocontrol function of *B. velezensis* LMY3-5

Compared with chemical preventive and control methods, biological control and prevention have attracted increasing attention because they offer green environmental protection and high safety to humans. *Pantoea* (Nunes et al., 2010; Usall et al., 2001), *Pseudomonas* (Lam et al., 1987), *Bacillus amyloliquefaciens* (Chen et al., 2016), *Trichoderma harzianum* (Zhang H. F. et al., 2022; Zhang Y. H. et al., 2022), *Fusicolla violacea* (Li et al., 2021) and *B. velezensis* (Ren et al., 2025) can inhibit the growth of plant pathogens.

Some of these bacteria have been successfully used for the biological control of soybean, grape, and other plant diseases. Compared with other beneficial antagonistic strains, *Bacillus* has better biocontrol application advantages. Coincidently, *Bacillus* is also the dominant flora in many plants. Notably, some *Bacillus* strains play a significant role in the prevention and control of plant pathogenic fungi or bacteria, such as *Fusarium graminearum* (Chan et al., 2003; Christopher et al., 2013), *Sclerotinia sclerotiorum* (Hou et al., 2006; Sun et al., 2017), *Xanthomonas oryzae* (Lin et al., 2002), *Bacillus vallismortis* (Xu et al., 2023), *Bacillus amyloliquefaciens* (Aleinikova et al., 2023) and *Bacillus velezensis* (Ren et al., 2025). In this study, LMY3-5 significantly inhibited the growth of *B. dothidea* and nd caused morphological alterations (e.g., mycelial distortion and fracture), thereby reducing the incidence of kiwifruit soft rot. These results suggest its potential as a biocontrol agent against this disease.

### 4.2 Genome mining analysis

Whole-genome sequencing can help determine the biocontrol potential of bacterial strains in agriculture (Grady et al., 2019., Pan et al., 2023). Extensive studies have demonstrated that whole-genome sequencing, proteomics, transcriptomics, metabolomics, and multi-omics integrated analysis have been increasingly applied in the field of biocontrol agents (Ding et al., 2021; Zhang H. F. et al., 2022; Zhang Y. H. et al., 2022). Exploring and predicting the types, structures, and biosynthetic pathways of microbial metabolites at the molecular level has become a mainstream approach for mining functional genes related to antimicrobial activity, discovering bioactive compounds, and investigating antimicrobial mechanisms. For instance, Li L. et al. (2023) and Li W. et al. (2023) utilized whole-genome and transcriptome analyses to predict 146 genes associated with carbohydrate-active enzymes (e.g., chitinases, proteases) and 35 putative gene clusters encoding antifungal metabolites in *Bacillus velezensis* Htq6, while also elucidating the stress response mechanisms of *Botrytis cinerea* to this biocontrol strain.

In this study, the genome of the LMY3-5 strain was sequenced, greatly enriching the genomic database of biocontrol *Bacillus* strains. The ANI value and cluster analysis of the genomes of *B. velezensis* SSF6, SCDB 291, and *B. amyloliquefaciens* LFB112 showed that LMY3-5 belonged to *B. velezensis* and had the closest relationship with strain SSF6. The genome of *Bacillus* contains genes encoding secondary metabolites and proteins that participate in various metabolic pathways. By performing comparative genomic data analysis using the NJ13 strain, eight different gene clusters related to secondary metabolite synthesis were identified in strain LMY3-5. Non-ribosomal peptide synthetases (NRPSs), polyketide synthases (PKSs), and hybrid enzymes synthesize cyclic lipopeptides such as surfactin, iturin, and fengycin, as well as polyketides and other antimicrobial metabolites. These compounds exert antifungal effects by inducing hyphal structural deformities, disrupting cell wall integrity and membrane permeability, and triggering oxidative stress responses, ultimately leading to pathogen death and disease suppression (Chen et al., 2009; Gong et al., 2015; Luo et al., 2015; Malik et al., 2024). Known surfactins exhibit antifungal and antifungal properties. In particular, surfactins can be embedded in bacterial cell membranes and dissolve phospholipid bilayers, resulting in the production of membrane pores and ion channels (Hamley, 2015; Stubbendieck and Straight, 2016). Surfactin exhibits significant antifungal activity against fungi. Its mechanism of action primarily involves the induction of intracellular ROS accumulation in fungal cells, leading to oxidative stress. Additionally, it reduces mitochondrial membrane potential, activates caspase-like proteases, and triggers chromatin condensation, indicating that fungal growth inhibition occurs via an apoptosis-like cell death pathways (Zhao et al., 2017; Cawoy et al., 2014; Chen et al., 2023). Notably, in one gene cluster, no homologous proteins with high similarity were identified, and it showed only 7% similarity with butirosin A or butirosin B synthesized in the saccharide pathway in *B. velezensis* FZB42. No homologous proteins were identified in the database for the other four gene clusters, indicating that LMY3-5 may produce new antifungal substances. In general, strain LMY3-5 has a rich antifungal substance synthesis gene cluster. Its antifungal activity against pathogenic fungi may be mainly derived from known substances with good antifungal activity; however, it produces other unknown metabolites that may also have antifungal activity.

### 4.3 Transcriptome analysis

Biocontrol strains regulate the growth of pathogens through the expression of antifungal-related genes and production of antifungal substances. Such as: Zhang H. F. et al. (2022) and Zhang Y. H. et al. (2022) obtained a high-quality genome sequence of *B. velezensis* GS-1 through multi-omics analysis and identified 13 gene clusters associated with secondary metabolite biosynthesis by integrating transcriptomic and metabolomic data. They predicted the potential production of antimicrobial lipopeptides, such as surfactin, iturin, and plantazolicin, and further confirmed the inhibitory effects of these lipopeptides against *Magnaporthe oryzae* along with their antagonistic mechanisms (Zhang H. F. et al., 2022; Zhang Y. H. et al., 2022). Su et al. (2021) analyzed the differentially expressed genes of *Sporisorium scitamineum* at different infection time points using transcriptomics and found that the number of differentially expressed genes peaked at 8 h post-infection. Fatty acid metabolism and peroxidases were identified as key metabolic pathways during the infection process of *S. scitamineum* (Su et al., 2021). Wang X. J. et al. (2021) performed whole-genome sequencing of Phomopsis phragmitis, the causative agent of kiwifruit soft rot, using the PacBio SMRT platform. They predicted 1,451 proteins associated with pathogen-host interactions and 558 virulence factors (Wang X. J. et al., 2021). To date, transcriptome sequencing has enabled the acquisition of extensive structural and functional information on genes. Although diverse secondary metabolites with antifungal activity have been discovered in various plant endophytic bacteria, and antimicrobial genes have been predicted, the regulatory mechanisms underlying their biosynthesis and their modes of antifungal action remain largely unknown. Further in-depth research is still required to elucidate their antimicrobial mechanisms.

In this study, we performed transcriptome sequencing after culturing *B. velezensis* LMY3-5 with *B. dothidea* for 72 h. Using the transcriptome data, we identified a total of 332 DEGs in the antagonistic LMY3-5 strain. The DEGs were mainly involved in benzoate degradation, starch and sucrose metabolism, PTS pathway, and biosynthesis of various other secondary metabolite pathways. The starch and sucrose metabolism pathways are mainly involved in the utilization of basic carbon and nitrogen sources in organisms. The biosynthesis of various antibiotics mainly involves the synthesis of related antifungal substances (such as lysozyme, dehydratase, and alanine-anti-capsular ligase). Thus, it can be deduced that this strain produces antifungal substances by initiating the metabolic synthesis pathways of related antifungal substances.

## 5 Conclusion

The biocontrol strain *B. velezensis* LMY3-5 demonstrates significant antagonistic activity against *B. dothidea*, the causal agent of kiwifruit soft rot, achieving 70.44% suppression efficacy in pathogen challenge assays. Genomic analysis revealed a 4.03 Mb circular chromosome harboring eight biosynthetic gene clusters (BGCs), including those encoding surfactin, fengycin, bacillaene, and bacillibactin, which collectively contribute to its antifungal potential. Transcriptomic profiling during coculture with *B. dothidea* identified 114 differentially expressed genes (DEGs), with upregulation of fengycin synthesis, siderophore production, and lysozyme-related genes, directly implicating these pathways in antifungal activity. Conversely, downregulation of flagellar assembly and chemotaxis genes suggests a metabolic shift toward secondary metabolite production during antagonism. Functional validation confirmed that lipopeptides (e.g., fengycin) and siderophores play pivotal roles in inhibiting fungal growth, likely through membrane disruption and iron competition. These findings provide molecular-level insights into LMY3-5’s biocontrol mechanisms and highlight its potential as a sustainable alternative to chemical fungicides for managing kiwifruit soft rot. Further field studies are warranted to translate these discoveries into practical agricultural applications.

## Data Availability

The data presented in the study are deposited in the NCBI GanBank repository, accession number CP196812 (https://www.ncbi.nlm.nih.gov/nuccore/CP196812.1).

## Footnotes

^1^ http://ccb.jhu.edu/software/glimmer/index.shtml

^2^ http://www.ezbiocloud.net/tools/ani

^3^ https://ggdc.dsmz.de/ggdc.php
